# Multi-resolution analysis for high-fidelity deconvolution microscopy

**DOI:** 10.1038/s41377-024-01654-4

**Published:** 2025-01-01

**Authors:** Baolei Liu, Fan Wang

**Affiliations:** School of Physics, Beihang University, 100191 Beijing, China

**Keywords:** Super-resolution microscopy, Imaging and sensing

## Abstract

A fidelity-ensured multi-resolution analysis deconvolution algorithm significantly enhances fluorescence microscopy’s resolution and noise control, enabling more accurate and detailed imaging for advanced biological research applications.

## Graphical abstract

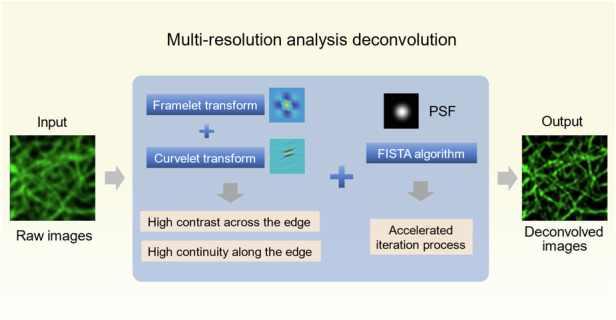

Fluorescence microscopy is a cornerstone in visualizing biological samples, enabling scientists to observe live cells and subcellular structures in detail^1^. Despite its critical role, traditional fluorescence microscopy often suffers from limitations imposed by optical blur and noise, which restricts imaging speed, duration, and resolution. High photon doses used to enhance imaging quality can also damage live cells, causing photobleaching and affecting cell viability. Deconvolution algorithms are essential in fluorescence microscopy because they improve image resolution and clarity by mathematically reversing the optical distortions introduced during image capture. These algorithms enable scientists to reconstruct high-quality images from noisy, blurred data^2–4^. However, traditional deconvolution methods often struggle with maintaining fidelity, particularly when pushing the limits of resolution enhancement. They can introduce artifacts, which may lead to misinterpretation of biological data. Therefore, developing a high-fidelity deconvolution algorithm that operates effectively under low signal-to-noise ratio (SNR) conditions is crucial for advancing fluorescence imaging.

In a recent publication in *eLight*^5^, the team from Peking University, China, and Airy Technologies Co., Ltd reported a new method, called multi-resolution analysis (MRA) to handle the inherent noise in fluorescence images, which offers a fidelity-ensured approach to image deconvolution and significantly enhances image quality by improving both resolution and noise control. This approach was successfully applied to wide-field, confocal, spinning disk confocal, structured illumination microscopy (SIM), stimulated emission depletion microscopy (STED), SoRa, and so on.

Unlike conventional variance-based regularizations that often entangle the continuity of biological structures with background noise, MRA distinguishes itself by focusing on two key attributes of fluorescence images: high contrast across edges and high continuity along edges. These features are critical in capturing the genuine characteristics of biological structures while effectively minimizing noise. As illustrated in Fig. 1, MRA can effectively detect and preserve these crucial features by utilizing the combination of framelet and curvelet transforms^6–8^. This leads to a significant improvement in the SNR of up to 10 dB higher than traditional methods. MRA can also provide a fidelity-ensured resolution improvement of up to two-fold, surpassing artifact-prone statistical approaches like Richardson-Lucy deconvolution. Additionally, MRA can be finely integrated with the fast iterative shrinkage-thresholding algorithm (FISTA) for its precise noise control and accelerated iteration^9^. The authors also developed an extension of MRA, called sectioning MRA (SecMRA), which incorporates a bias thresholding mechanism to attenuate fluorescence background. SecMRA has a remarkable computational sectioning ability for cases with heavy background conditions.

**Fig. 1:**
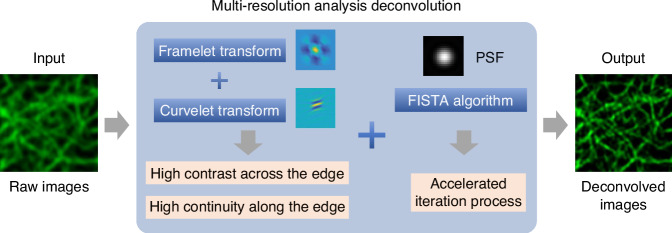
Schematic illustration of the multi-resolution analysis deconvolution algorithm

The implications of MRA and SecMRA are far-reaching, offering solutions to some of the most pressing challenges in biomedical imaging. The methods have demonstrated superior performance across various imaging modalities, including diffraction-limited and super-resolution microscopy. For example, MRA successfully resolved ~60 nm resolution features with ensured fidelity using SIM, a challenging task for traditional methods. MRA has been demonstrated with fast and long-term live-cell imaging to capture the dynamics of the endoplasmic reticulum, with an imaging speed of ~417 Hz and a duration time of ~10 min. The authors also demonstrated the effectiveness of SecMRA with low‑toxicity, long-term fluorescence imaging of organelle interactions with a duration of more than 30 minutes. This ability to maintain high fidelity over extended imaging periods with low photobleaching and phototoxicity opens new avenues for studying dynamic biological processes in real-time.

The development of MRA represents a significant step forward in the quest for more reliable and accurate fluorescence microscopy. Moreover, it can enhance commercial imaging systems, such as CSU-W1 SoRa and LiveSR microscopes, providing researchers with more accessible and accurate study tools. With its open-access MATLAB source code and raw fluorescence image data, the team encourages the broader scientific community to adopt and refine MRA, ensuring widespread adoption and further innovation. Future works may focus on the integration of MRA with modified physical model models and machine learning approaches to further enhance its performance for high-fidelity, high-resolution, high-speed and long-duration fluorescence imaging.
